# Associations of Body Mass Index, Weight Change, Physical Activity, and Sedentary Behavior With Endometrial Cancer Risk Among Japanese Women: The Japan Collaborative Cohort Study

**DOI:** 10.2188/jea.JE20200145

**Published:** 2021-12-05

**Authors:** Hiromi Miyata, Kokoro Shirai, Isao Muraki, Hiroyasu Iso, Akiko Tamakoshi

**Affiliations:** 1 Public Health, Department of Social Medicine, Graduate School of Medicine, Osaka University, Osaka, Japan; 2 Department of Public Health Medicine, Faculty of Medicine, University of Tsukuba, Ibaraki, Japan; 3 Department of Public Health, Faculty of Medicine and Graduate School of Medicine, Hokkaido University, Hokkaido, Japan

**Keywords:** endometrial carcinoma, body mass index, exercise, sedentary behavior, cohort study

## Abstract

**Background:**

The impact of weight change, physical activity, and sedentary behavior on endometrial cancer risk among the Asian population is uncertain. We investigated the association of those factors with endometrial cancer risk among Japanese women with a low body mass index level.

**Methods:**

We performed a large-scale nationwide cohort study consisting of 33,801 female participants aged 40–79 years. The Cox proportional hazards model was used to calculate the hazard ratios (HRs) and 95% confidence intervals (CIs) of incident endometrial cancer.

**Results:**

The mean body mass index of participants was 22.8 kg/m^2^. During a median follow-up of 14.8 years, 79 participants developed endometrial cancer. After adjustment for potential confounding factors, body mass index over 23.0 kg/m^2^ was linearly associated with the risk of endometrial cancer. The HR per 5 kg/m^2^ increase was 1.80 (95% CI, 1.28–2.54). Weight increment ≥+5 kg since age 20 was associated with an increased risk of endometrial cancer compared to a weight change of −5 to <+5 kg (multivariable HR 1.96; 95% CI, 1.12–3.40). Compared with females who were mainly sitting at the worksite, those who were mainly standing and moving were at lower risk; the multivariable HRs were 0.79 (95% CI, 0.39–1.59) and 0.46 (95% CI, 0.22–0.97), respectively (*P* for trend = 0.042). Hours of physical exercise, daily walking, and TV viewing were not associated with endometrial cancer risk.

**Conclusions:**

Overweight and weight gain were positively associated with the risk of endometrial cancer, while worksite physical activity was inversely associated with the risk.

## INTRODUCTION

Endometrial cancer is the sixth most common type of cancer worldwide in women, following breast, colorectal, lung, cervical/uterine, and thyroid.^1^ The age-adjusted incidence rate of endometrial cancer in Japan has increased during the past 10 years from 8.4/100,000 in 2004 to 16.0/100,000 in 2014.^2^ The average annual percent change in incidence rate between 2001 and 2010 was appreciable in Japan and it was the second fastest increase among 42 countries.^3^

Various risk factors for endometrial cancer have been addressed. Obesity, nulliparity, unopposed estrogen therapy, late menopause, tamoxifen therapy, and diabetes, and those associated with unopposed estrogen stimulation were associated with an increased risk of endometrial cancer,^4^^,^^5^ whereas breastfeeding,^6^ oral contraceptive use,^7^ and physical activity^8^ were associated with a decreased risk. These findings have come primarily from Western populations but not from Asian populations.^9^^–^^11^ Therefore, the generalization of such findings to other ethnic groups requires confirmation.

The definition of obesity in Asian populations is different from that used in Western populations. The World Health Organization (WHO) proposed body mass index (BMI) guidelines for Asia-Pacific populations as follows: underweight (BMI <18.5 kg/m^2^), normal weight (BMI 18.5–22.9 kg/m^2^), overweight (BMI 23.0–24.9 kg/m^2^), obese (BMI 25.0–29.9 kg/m^2^), and morbidly obese (BMI ≥30.0 kg/m^2^),^12^ which are different from the traditional cut-offs of obesity (BMI ≥30.0 kg/m^2^) and overweight (BMI 25.0–29.9 kg/m^2^) used for Western populations. Actually, in Japan, approximately 20% of adults have a BMI ≥25.0 kg/m^2^,^13^ which is much lower than in other high-income countries (eg, 57% in the United Kingdom, 60% in New Zealand, and 62% in the United States).^14^

Weight increase in adulthood has also been associated with endometrial cancer risk.^15^^,^^16^ Whether the weight increase itself, independent of being overweight, is associated with endometrial cancer risk or not is unclear.^17^

Length of sitting time among Japanese people (median ≥360 min/day) has been reported as being the highest of 20 countries surveyed.^18^ There is no publication on the association of physical activity and sedentary behavior with endometrial cancer risk among Asian populations.

Obesity, physical activity, and sedentary behavior are mutable factors. Therefore, the aim of this study was to determine whether BMI, weight change, physical activity, and sedentary behavior were associated with endometrial cancer in Japanese participants aged 40–79 years.

## METHODS

### Study design, setting and participants

The Japan Collaborative Cohort Study for Evaluation of Cancer Risk (JACC Study) was initiated in 1988, and enrolment continued until the end of 1990. Details of our research have been described in previous publications.^19^ Briefly, a total of 110,585 (46,395 men and 64,190 women) inhabitants aged 40–79 years from 45 areas throughout Japan participated in municipal health screening examinations. They completed self-administered questionnaires at enrolment. Participants in this study were limited to 24 study areas where the incidence of cancer could be ascertained and recorded in the cancer registry. Of 38,613 original female cohort participants, 4,812 were excluded because they had a history of cancer (708) or uterine surgery (4,583). Consequently, 33,801 participants were evaluated in this study.

Study participants were informed in writing about the objectives and methods of the JACC study. In some areas, consent was obtained from the representative in those areas. The institutional review board of Nagoya University School of Medicine approved the study protocol.

### Outcomes

The incidence of cancer could be ascertained in 24 study areas where population-based cancer registries or local major hospital records were available for review and coded according to the tenth revision of the International Classification of Disease (ICD-10). The primary outcome of interest in the present study was the incidence of endometrial cancer (ICD-10: C54). The date and cause of death were confirmed using death certificates. Participants who moved away from the study area during the study period were treated as censored cases. The median follow-up period for cancer incidence surveys was 14.8 years, as follow-up surveys were discontinued in some study areas before 2009.

### Variables

The self-administered questionnaires at enrolment contained the following questions: the presence or absence of medical history (cancer, operation, diabetes, hypertension, and others); body size (weight, height, weight at age 20); reproductive factors (age at menarche, age at menopause, parity); smoking status (never, past, or current smoker); alcohol consumption (never, past, or current drinker); lifestyle (hours of physical exercise, daily walking, television (TV) viewing, sleep duration); occupation (job type, physical activity at work, workplace). Validity and reliability of questions about physical activity in this study were reported in the previous study.^20^

BMI was calculated as weight/(height)^2^. We categorized BMI into five groups: <18.5, 18.5 to <23.0, 23.0 to <25.0, 25.0 to <27.5, and ≥27.5 kg/m^2^, based on the WHO BMI classification. Weight difference was calculated as baseline weight minus weight at age 20, then categorized into three groups: <−5, −5 to <+5, ≥+5 kg.

We also categorized each variable as follows: age at menarche (tertiles: ≤13, 14, ≥15 years old), menstrual presence (premenopausal, postmenopausal), parity (0, 1 or 2, ≥3), smoking status (never, past, current smoker), alcohol consumption (never, past, current drinker), occupational activity was classified according to the position during work (mainly sitting, mainly standing, moving), hours of physical exercise (never, 1–2, ≥3 hours/week), daily walking (<0.5, 0.5 to <1, ≥1 hour/day) and TV viewing (<1, 1 to <2, 2 to <3, 3 to <4, ≥4 hours/day). The above cut-points for categorization of each variable were decided based on the hypothesized biological effects on endometrial cancer risk and the frequency distribution within the cohort.

### Statistical analysis

Using BMI 18.5 to <23.0 kg/m^2^ and weight difference −5 to <+5 kg as reference categories, age-adjusted hazard ratios (HRs) and their 95% confidence intervals (CIs) were calculated using Cox proportional analysis in the first model. The second model was adjusted for age, BMI at age 20, history of hypertension, history of diabetes, age at menarche, menstrual presence, parity, smoking status, alcohol consumption, occupational activity, hours of physical exercise, daily walking, and TV viewing. We encoded missing indications itself as a single category and analyzed.

Stratified analyses were conducted to explore whether the effects of weight change on endometrial cancer were modified by baseline BMI. We stratified participants into two groups based on baseline BMI of 23.0 kg/m^2^. Using a weight difference of −5 to <+5 kg as the reference category, age-adjusted HRs and their 95% CIs were calculated using Cox proportional analysis in the first model. The second model was adjusted further for history of hypertension, history of diabetes, age at menarche, menstrual presence, parity, smoking status, alcohol consumption, occupational activity, hours of physical exercise, daily walking TV viewing.

Regarding variables related to physical activity, using mainly sitting at work, hours of physical exercise of 1–2 hours/week, daily walking of 0.5 to <1 hour/day, and TV viewing of 1 to <2 hours/day as reference categories, age-adjusted HRs and their 95% CIs were calculated using Cox proportional analysis. The second model was adjusted further for baseline BMI, weight change since age 20, history of diabetes, history of hypertension, age at menarche, menstrual presence, parity, smoking status, alcohol consumption, occupational activity, hours of physical exercise, daily walking, and TV viewing. In the analysis of occupational activity, unemployed women (*n* = 5,938) were excluded from the analysis.

Trend tests were estimated on the median value of each category or the representative value and entered as a continuous term in the regression models.

All statistical analyses were performed using SAS 9.4 (SAS Institute Inc., Cary, NC, USA), and a *P* < 0.05 was considered statistically significant.

## RESULTS

Table 1 shows the baseline characteristics of participants based on baseline BMI and weight change since age 20. The mean age of participants was 58.1 years and 20.6% of participants were overweight and obese (≥25.0 kg/m^2^) at baseline. Weight change since age 20 was incremented with increased baseline BMI. Mean values for age at menarche, age at menopause, parity and TV viewing time did not differ among the categories of BMI and weight change. The proportions of positive histories of hypertension and diabetes were higher with increased BMIs. Current smoker was more common among persons in the lowest and highest BMI groups. Current alcohol consumption was more common among persons with normal baseline BMI and in the highest weight gain group. We observed a higher proportion of physical exercise ≥5 hours/week and walking ≥1 hour/day among the lower BMI and weight loss groups.

**Table 1.** Participants' baseline characteristics according to baseline body mass index and weight change since age 20

	Total	Baseline body mass index, kg/m^2^						Weight change since age 20, kg			
	
		<18.5	18.5 to <23.0	23.0 to <25.0	25.0 to <27.5	≥27.5	*P* for trend	<−5	−5 to <+5	≥+5	*P* for trend
Number at risk	33,801	4,085	15,488	7,259	4,737	2,232		11,259	11,648	10,894	
Age^*^, years	58.1	63.5	57.3	57.1	58.0	57.9	<0.001	62.5	56.4	56.4	<0.001
Body mass index^*^, kg/m^2^	22.8	17.4	21.1	23.9	26.1	29.6	<0.001	21.8	21.8	24.9	<0.001
Weight change since 20 years old^*^, kg	2.6	−5.2	−0.5	4.6	8.4	14.4	<0.001	−9.3	−0.5	10.3	<0.001
Age at menarche^*^, years	15.0	15.4	15.0	14.9	14.8	14.8	<0.001	15.3	14.9	14.7	<0.001
Age at menopause^*^, years	49.2	48.7	49.3	49.3	49.4	49.1	<0.001	48.9	49.3	49.4	<0.001
Parity^*^	2.7	2.8	2.6	2.7	2.8	2.9	<0.001	2.9	2.6	2.6	0.100
History of hypertension, *n* (%)	7,261 (21.48)	770 (18.9)	2,587 (16.7)	1,629 (22.4)	1,426 (30.1)	849 (38.0)	<0.001	2,568 (22.8)	1,966 (16.9)	2,727 (25.0)	<0.001
History of diabetes, *n* (%)	1,329 (3.9)	163 (4.0)	54 (3.5)	280 (3.9)	192 (4.1)	146 (6.5)	0.002	526 (4.7)	370 (3.2)	433 (4.0)	0.031
Current smoking, *n* (%)	1,473	233 (6.8)	652 (4.7)	286 (4.3)	174 (4.0)	128 (6.4)	0.006	517 (5.3)	468 (4.4)	488 (4.9)	0.003
Current alcohol consumption, *n* (%)	7,360	686 (19.1)	3,485 (23.9)	1,682 (24.5)	1,043 (23.4)	464 (22.3)	0.354	2,029 (19.9)	2,646 (24.0)	2,685 (26.0)	<0.001
Television viewing time^*^, hours/day	2.9	3.0	2.8	2.9	3.0	3.3	<0.001	3.0	2.7	3.0	<0.001
Physical exercise, ≥5 hours/week, *n* (%)	1,230	141 (4.3)	577 (4.2)	262 (4.1)	183 (4.4)	67 (3.4)	0.912	410 (5.2)	449 (4.1)	371 (3.6)	0.440
Walking time, ≥1 hour/day, *n* (%)	13,803	1,583 (49.0)	6,649 (50.7)	2,934 (49.4)	1,847 (48.8)	790 (47.4)	0.030	3,977 (51.2)	5,267 (50.8)	4,559 (47.5)	<0.001
Activity at occupation, mostly sitting, *n* (%)	9,913	1,274 (45.1)	4,702 (40.4)	2,043 (38.9)	1,294 (39.3)	600 (40.3)	<0.001	2,836 (41.1)	5,390 (58.5)	3,487 (41.6)	0.021

^*^: mean value.

With follow-up for a median of 14.8 years, 79 of the 33,801 healthy Japanese women participants developed endometrial cancer. Among the study participants, 6,060 died, 1,958 moved out of the study area, and 5 were lost to follow up during the study period.

The HRs (95% CIs) of incident endometrial cancer according to baseline BMI are shown in Table 2. There were significant positive associations of BMI with risk of endometrial cancer. In the second model, multivariable HRs of endometrial cancer with reference to a baseline BMI of 18.5 to <23.0 kg/m^2^ were 0.59 (95% CI, 0.14–2.53) for a BMI of <18.5 kg/m^2^, 1.93 (95% CI, 1.05–3.53) for a BMI of 23.0 to <25.0 kg/m^2^, 1.87 (95% CI, 0.93–3.74) for a BMI of 25.0 to <27.5 kg/m^2^, and 2.80 (95% CI, 1.25–6.30) for a BMI ≥27.5 kg/m^2^. We observed statistically significant trends in all models. The HR per 5-unit (kg/m^2^) increase was 1.80 (95% CI, 1.28–2.54) in model 2. We did sensitivity analysis excluding 15 cases of endometrial cancer that occurred in the follow-up duration within 2 years. The positive associations of BMI with the risk of endometrial cancer did not change (not shown in table).

**Table 2.** Hazard ratios (95% confidence intervals) of incident endometrial cancer according to baseline body mass index

	Baseline body mass index, kg/m^2^					*P* for trend	Hazard ratios per 5-unit (kg/m^2^) increase

	<18.5	18.5 to <23.0	23.0 to <25.0	25.0 to <27.5	≥27.5		
Number at risk	4,085	15,488	7,259	4,737	2,232		
Person years	54,179	207,409	98,054	64,861	31,031		
Number of cases	8	24	22	13	12		
Model 1^a^	1.29 (0.57–2.91)	1	1.94 (1.09–3.46)	1.73 (0.88–3.40)	3.34 (1.67–6.69)	<0.001	1.66 (1.25–2.20)
Model 2^b^	0.59 (0.14–2.53)	1	1.93 (1.05–3.53)	1.87 (0.93–3.74)	2.80 (1.25–6.30)	<0.001	1.80 (1.28–2.54)

^a^Model 1 adjusted for age.

^b^Model 2 adjusted further for body mass index at age 20, history of diabetes, history of hypertension, age at menarche, menstrual presence, parity, smoking status, alcohol consumption, occupational activity, hours of physical exercise, walking, and television viewing.

As for weight change since age 20 (Table 3), a body weight increment of >+5 kg was associated with an increased risk of endometrial cancer compared with a weight change of −5 to <+5 kg (the multivariable HR was 1.96; 95% CI, 1.12–3.40), while a decrease in body weight was not associated with a lower risk in the second model. We observed statistically significant trends in both models. We did sensitivity analysis excluding 15 cases of endometrial cancer that occurred in the follow-up duration within two years. The positive associations of body weight increment with the risk of endometrial cancer did not change (not shown in table).

**Table 3.** Hazard ratios (95% confidence intervals) of incident endometrial cancer according to weight change since age 20

	Weight change since age 20, kg			*P* for trend

	<−5	−5 to <+5	≥+5	
Number at risk	11,259	11,648	10,894	
Person years	145,986	161,502	148,047	
Number of cases	17	22	40	
Model 1^a^	0.80 (0.42–1.54)	1	2.00 (1.19–3.36)	0.001
Model 2^b^	0.78 (0.30–2.02)	1	1.96 (1.12–3.40)	0.001

^a^Model 1 adjusted for age.

^b^Model 2 adjusted further for body mass index at age 20, history of diabetes, history of hypertension, age at menarche, menstrual presence, parity, smoking status, alcohol consumption, occupational activity, hours of physical exercise, walking, and television viewing.

Table 4 shows the results of stratified analyses using baseline BMI. There were significant positive associations between weight increment and risk of endometrial cancer in the baseline BMI of <23.0 kg/m^2^ but not in the baseline BMI of ≥23.0 kg/m^2^. The respective multivariable HRs for weight change ≥+5 kg versus weight change −5 to <+5 kg were 2.42 (95% CI, 1.01–5.79) and 1.14 (95% CI, 0.56–2.35).

**Table 4.** Hazard ratios (95% confidence intervals) of incident endometrial cancer according to weight change, stratified by baseline body mass index

	Baseline body mass index <23.0 kg/m^2^				Baseline body mass index ≥23.0 kg/m^2^			
	
	Weight change since age 20, kg			*P* for trend	Weight change since age 20, kg			*P* for trend
	
	<−5	−5 to <+5	≥+5		<−5	−5 to <+5	≥+5	
Number at risk	8,168	8,499	2,906		3,091	3,149	7,988	
Person years	106,164	117,198	38,227		39,822	44,304	109,820	
Number of cases	11	12	9		6	10	31	
Model 1^a^	0.93 (0.40–2.20)	1	2.33 (0.98–5.56)	0.078	0.66 (0.24–1.81)	1	1.27 (0.62–2.59)	0.178
Model 2^b^	1.09 (0.45–2.62)	1	2.42 (1.01–5.79)	0.135	0.80 (0.28–2.29)	1	1.14 (0.56–2.35)	0.467

^a^Model 1 adjusted for age.

^b^Model 2 adjusted further for history of diabetes, history of hypertension, age at menarche, menstrual presence, parity, smoking status, alcohol consumption, occupational activity, hours of physical exercise, walking, and television viewing.

The HRs (95% CIs) of incident endometrial cancer according to occupational activity are shown in Table 5. There was an inverse association of occupational activity and endometrial cancer risk. The multivariable HRs of endometrial cancer with reference to mainly sitting were 0.79 (95% CI, 0.39–1.59) for mainly standing and 0.46 (95% CI, 0.22–0.97) for moving.

**Table 5.** Hazard ratios (95% confidence intervals) of incident endometrial cancer according to occupational activity

	Activity at occupation			*P* for trend

	Mainly sitting	Mainly standing	Moving	
Number at risk	6,726	4,648	7,183	
Person years	98,570	59,414	96,748	
Number of cases	24	12	11	
Model 1^a^	1	0.78 (0.39–1.57)	0.45 (0.22–0.92)	0.028
Model 2^b^	1	0.79 (0.39–1.59)	0.46 (0.22–0.97)	0.042

^a^Model 1 adjusted for age.

^b^Model 2 adjusted further for baseline body mass index, weight change since age 20, history of diabetes, history of hypertension, age at menarche, menstrual presence, parity, smoking status, alcohol consumption, hours of physical exercise, walking, and television viewing.

Hours of physical exercise, walking and TV viewing were not associated with the risk of endometrial cancer (Table 6).

**Table 6.** Hazard ratios (95% confidence intervals) of incident endometrial cancer according to the hours of physical exercise, walking and television viewing

	Physical exercise, hour/week			*P* for trend

	Never	1 to 2	≥3	
Number at risk	22,335	4,189	2,822	
Person years	307,497	56,188	38,022	
Number of cases	52	13	5	
Model 1^a^	1.22 (0.71–2.11)	1	0.86 (0.32–2.34)	0.883
Model 2^b^	0.77 (0.42–1.43)	1	0.58 (0.21–1.64)	0.775

	Daily walking, hour/day			*P* for trend

	<0.5	0.5 to <1	≥1	
Number at risk	2,769	11,164	13,803	
Person years	37,302	141,860	188,400	
Number of cases	9	26	32	
Model 1^a^	1.49 (0.72–3.09)	1	1.05 (0.65–1.68)	0.539
Model 2^b^	1.37 (0.63–2.95)	1	1.07 (0.63–1.81)	0.712

	Television viewing, hour/day					*P* for trend

	<1	1 to <2	2 to <3	3 to <4	≥4	
Number at risk	3,999	4,728	8,481	8,056	8,537	
Person years	46,318	67,533	119,762	112,222	109,701	
Number of cases	3	12	24	16	24	
Model 1^a^	0.35 (0.10–1.24)	1	1.13 (0.57–2.26)	0.81 (0.38–1.71)	1.24 (0.61–2.52)	0.124
Model 2^b^	0.50 (0.13–1.92)	1	1.07 (0.54–2.15)	0.72 (0.34–1.53)	1.05 (0.51–2.15)	0.635

^a^Model 1 adjusted for age.

^b^Model 2 adjusted further for baseline body mass index, weight change since age 20, history of diabetes, history of hypertension, age at menarche, menstrual presence, parity, smoking status, alcohol consumption, occupational activity, hours of physical exercise, walking, and television viewing.

## DISCUSSION

In this large cohort study, we found that a BMI ≥23.0 kg/m^2^ at baseline was associated with an increased risk of incident endometrial cancer among Japanese women. Our results were consistent with findings from previous studies, including cohort studies and meta-analyses.^21^^–^^23^ Most previous studies set the cut-off point for BMI at ≥25.0 kg/m^2^ for overweight and ≥30.0 kg/m^2^ for obesity. In our cohort, only 623 participants (1.8%) had a BMI ≥30.0 kg/m^2^, and 6,346 (18.8%) had a BMI of 25.0 to <30.0 kg/m^2^. Therefore, we set the cut-off points at 23.0, 25.0, and 27.5 kg/m^2^ based on WHO BMI guidelines for Asia-Pacific populations. Using a baseline BMI of 18.5 to <23.0 kg/m^2^ as the reference, the multivariable HRs for baseline BMIs of 23.0 to <25.0 kg/m^2^, 25.0 to <27.5 kg/m^2^, and ≥27.5 kg/m^2^ were 1.93 (95% CI, 1.05–3.53), 1.87 (95% CI, 0.93–3.74), and 2.80 (95% CI, 1.25–6.30), respectively. Our research showed that a baseline BMI of 23.0 to <25.0 kg/m^2^ was associated with a 2-fold higher risk of endometrial cancer and the risk was 3-fold higher for participants with a baseline BMI ≥27.5 kg/m^2^.

We also found that a weight increment ≥+5 kg since age 20 was associated with a 2-fold higher risk of endometrial cancer, which was consistent with the findings of previous cohort studies performed in California and Europe.^15^^,^^16^ A prospective study of 28,418 postmenopausal women living in California, in which 42.5% of participants had a BMI ≥25.0 kg/m^2^, showed that weight gain since age 18 >11.4 kg was associated with a 3-fold increased risk of endometrial cancer compared with a weight change of −11.4 to <+11.4 kg among never users of hormone replacement therapy, while weight gain was not associated with endometrial cancer risk among users. A prospective study of 223,008 women aged between 35 and 70 in Europe, in which 45.1% of participants had BMI ≥25.0 kg/m^2^, showed that adult weight gain since age 20 ≥20 kg was associated with a 2-fold increased risk of endometrial cancer compared with a weight change of −3 to <+3 kg.

No studies have examined whether weight gain since age 20 was associated with endometrial cancer risk among women with a low BMI. Our stratified analysis showed that weight gain since age 20 was associated with an increased risk of endometrial cancer in the groups with a baseline BMI <23.0 kg/m^2^. Therefore, our results suggest that the avoidance of weight gain may be beneficial for prevention of endometrial cancer even when the baseline BMI was in the normal range.

The detailed biological mechanisms that explain why obesity and weight gain lead to cancer are still lacking. Recent research showed that obesity leads to the development of dysfunctional adipose tissue, which produces abundant levels of proinflammatory cytokines, sex hormones and lipid metabolites, along with altered adipokine profiles.^24^ Obesity-associated systemic metabolic changes, such as hyperinsulinemia and hyperglycaemia, also contribute to a tumor-permissive environment.^25^ A cross-sectional study of 399 Japanese municipal employees aged ≥30 years showed positive associations between blood adipokine concentrations and weight gain from the age of 20 years after further adjustment for current BMI.^26^ Thus, even among women with a low BMI, adult weight gain contributes to increase the risk of adiposity-related cancer by increasing adipokine concentrations.

It has been reported that sedentary behavior was associated with an increased risk of endometrial, breast, and colorectal cancer.^27^^,^^28^ In our research, compared with mainly sitting at work, mainly standing and moving at work was associated with a lower risk even after adjustment for confounding factors, including BMI. A previous case-control study of 1,572 women (542 cases and 1,032 controls) aged 30–79 years in Canada showed that the risk of endometrial cancer with lower occupational activity (≤2.4 MET-hour/week/year versus >11.3 MET-hour/week/year) was 1.28 (95% CI, 0.89–1.83).^29^ That case-control study and a meta-analysis of 14 case-control, one case-cohort, and 18 prospective cohort studies^8^ indicated that even total physical activity of light intensity compared with vigorous physical activity had a more beneficial effect on the risk of endometrial cancer; the summary odds ratio was 0.65 (95% CI, 0.49–0.86) for light intensity and 0.80 (95% CI, 0.72–0.90) for vigorous activity. Our result that occupational activity was associated with a decreased risk of endometrial cancer was consistent with findings from those previous studies.^8^^,^^29^

The effects of physical activity or sedentary behavior on the immune system, epigenetics, protein translation and microbiome, in relation to cancer development are under active investigation.^30^^,^^31^ Physical activity can reduce estradiol concentrations,^32^ which were positively associated with endometrial cancer risk.^33^ Long sitting time could increase levels of inflammatory factors, such as tumor necrosis factor-A, interleukin-6, and leptin, known risk predictors for cancer.^34^

In our study, hours of physical exercise, walking, and TV viewing were not associated with the risk of endometrial cancer. A previous prospective cohort study of 71,570 women in the Nurses’ Health Study^35^ investigated the association between recreational physical activity and risk of endometrial cancer. The simple update assessment every 2–4 years, reflecting recent activity, was inversely associated with risk of endometrial cancer, but baseline recreational activity was not, suggesting a short-term protective effect of physical activity on the risk. Our results that hours of physical exercise, walking, and TV viewing were not associated with the risk of endometrial cancer, could be due to use of non-updated baseline data.

The strengths of our study include its prospective design, the active endpoint determination using a cancer registry, and its large sample size of participants throughout Japan. Additionally, the information on potential confounders for endometrial cancer was collected at baseline and adjusted in the multivariable analyses.

The limitations of this study were as follows. First, the number of endometrial cancer cases was small (*n* = 79); therefore, the lack of association of physical exercise and walking with endometrial cancer risk could be because of the low statistical power. Second, we collected data at baseline only and that information was not updated; therefore, subsequent lifestyle changes could not be taken into account. Weight at age 20 in the baseline survey relied on the participants’ memories. A previous study about the accuracy of long-term recall of past body weight in Japanese showed that recalled weight around age of 25 strongly correlated with measured weight at age 25 (*r* = 0.849).^36^ Therefore, a recall bias regarding past body weight would not affect our results materially. Third, while previous reports showed that the association of BMI and risk of endometrial cancer was stronger in postmenopausal than premenopausal women,^16^ we did not conduct a stratified analysis according to menopausal status because the number of endometrial cancer cases was small. We, however, adjusted for menstrual status. Fourth, we did not have the data on breastfeeding, oral contraceptive use, and hormone replacement therapy. Previous reports showed that breastfeeding^6^ and oral contraceptive use^7^ were associated with a decreased risk of endometrial cancer. A 10-year prospective study of 28,418 American women showed that BMI was positively associated with endometrial cancer risk among never users of hormone replacement therapy, but that a positive association was not evident among hormone users.^15^ In 1985, over 90% of Japanese women had experienced breastfeeding for up to 1 month after the birth of their infant.^37^ In Japan, oral contraceptives were approved in 1999. The prevalence of hormone replacement therapy was only 2.5% among women aged 45–64 years in 1992.^38^ Therefore, breastfeeding, oral contraceptives, and hormone replacement therapy were unlikely to affect our results.

In conclusion, overweight and weight gain since age 20 were associated with a higher risk of endometrial cancer, while being occupationally active was associated with a lower risk of endometrial cancer among Japanese women whose average BMI was low.
